# Transcription Regulation of the Human Telomerase Reverse Transcriptase (*hTERT*) Gene

**DOI:** 10.3390/genes7080050

**Published:** 2016-08-18

**Authors:** Muhammad Khairul Ramlee, Jing Wang, Wei Xun Toh, Shang Li

**Affiliations:** 1 Cancer and Stem Cell Biology Program, Duke-NUS Graduate Medical School, Singapore 169857, Singapore; khairul@u.duke.nus.edu (M.K.R.); wang.jing@u.duke.nus.edu (J.W.); wei.xun@u.duke.nus.edu (W.X.T.); 2 Department of Physiology, Yong Loo Lin School of Medicine, National University of Singapore, Singapore 117597, Singapore

**Keywords:** telomerase, telomere, transcription regulation, promoter, mutation

## Abstract

Embryonic stem cells and induced pluripotent stem cells have the ability to maintain their telomere length via expression of an enzymatic complex called telomerase. Similarly, more than 85%–90% of cancer cells are found to upregulate the expression of telomerase, conferring them with the potential to proliferate indefinitely. Telomerase Reverse Transcriptase (TERT), the catalytic subunit of telomerase holoenzyme, is the rate-limiting factor in reconstituting telomerase activity in vivo. To date, the expression and function of the human Telomerase Reverse Transcriptase (*hTERT*) gene are known to be regulated at various molecular levels (including genetic, mRNA, protein and subcellular localization) by a number of diverse factors. Among these means of regulation, transcription modulation is the most important, as evident in its tight regulation in cancer cell survival as well as pluripotent stem cell maintenance and differentiation. Here, we discuss how *hTERT* gene transcription is regulated, mainly focusing on the contribution of trans-acting factors such as transcription factors and epigenetic modifiers, as well as genetic alterations in *hTERT* proximal promoter.

## 1. Introduction

The ends of human chromosomes are capped by telomeres which protect the chromosome termini from degradation, end-to-end fusion and recombination [1,2]. Telomeres are long stretches of 5′-TTAGGG-3′ DNA repeats which end in single-stranded 3′ G-rich overhangs [3,4]. The telomeric DNA repeats are bound by shelterin protein complexes consisting of Telomeric Repeat Factor I and II (TRF1, TRF2), Repressor/Activator Protein I (RAP1), TRF1-Interacting Nuclear Protein 2 (TIN2), Tripeptidyl Peptidase I (TPP1) and Protection of Telomeres I (POT1) that distinguish naturally occurring chromosomal ends from DNA double-strand breaks [2]. Telomeric DNA repeats are synthesized by telomerase, a reverse transcriptase. Human core telomerase consists of at least two essential subunits, the protein subunit, human Telomerase Reverse Transcriptase (hTERT), and the RNA subunit, human Telomerase RNA (hTR) [5,6]. Telomerase activity is generally limited by the expression of hTERT, and is barely detected in most human adult somatic tissues, except in germ cells and some stem cells [5,6,7,8,9]. In cells lacking telomerase activity, about 50–200 bp of telomeric DNA repeats are lost during each cell division due to incomplete replication by DNA polymerase, and end processing [4,10].

Adult human somatic cells have limited telomere lengths ranging from 7 to 12 kb [11]. Therefore, progressive telomere shortening may function as an internal clock that determines the replicative capacity of normal human somatic cells [12]. When telomeres shorten to a critical limit, they become uncapped and trigger Ataxia Telangiectasia Mutated (ATM)- and/or Ataxia Telangiectasia and Rad3-Related Protein (ATR)-dependent DNA damage signaling cascades [13,14,15]. Through the downstream transducer kinases, Checkpoint kinase I and II (CHK1 and CHK2), the uncapped chromosomal ends are marked by distinct telomere dysfunction-induced foci (TIFs) [13]. As few as five dysfunctional telomeres are sufficient to trigger irreversible cell cycle arrest, termed replicative senescence, in primary human fibroblast cells [14,15,16]. As a result, normal somatic cells can only proliferate for a limited number of passages that is pre-set by their telomere length [16]. In contrast to pluripotent stem cells whose telomere length is sustained, telomerase activity in tissue-specific progenitor/stem cells is not sufficient for complete telomere maintenance [17]. Consequently, telomere length in these tissue-specific progenitor/stem cells also progressively shortens. This decrease in length limits the proliferative capacity of tissue-specific progenitor/stem cells, and contributes to normal human aging [18]. Therefore, the process of resetting telomere length during early embryogenesis is necessary to ensure sufficient telomere reserves for cell integrity during human development and aging [19].

Defects in genes that regulate telomere length homeostasis may lead to various diseases, collectively termed telomeropathies [20,21]. Patients display diverse symptoms such as premature aging and increased risk of cancer, highlighting the importance of telomere homeostasis in human health. Mutations in telomerase and telomere maintenance genes have been found in patients with telomeropathies [20,21,22], which results in decreased telomerase activity and accelerated telomere shortening. These genes control telomerase ribonucleoprotein maturation, assembly, recruitment, and engagement, revealing their importance in telomere length homeostasis. To add, telomerase knockout mouse models show remarkable similarity to the human disease phenotypes [23,24,25]. Strategies that promote the expression of *hTERT* and restore telomere length homeostasis could potentially delay the clinical onset of these diseases as well as normal aging in humans.

On the other hand, telomerase activity is highly elevated in 85%–90% of human cancers and over 70% of immortalized human cell lines [7,26]. This is consistent with telomerase conferring a strong selective advantage for continued growth of malignant cells [27]. These observations suggest that telomere maintenance is essential for cancer cell immortalization and that it may be possible to inhibit cancer growth by interfering with telomerase activity.

Expression and function of *hTERT* gene are known to be regulated at various molecular levels. However, the transcription of *hTERT* has been suggested to be the dominant step in the regulation of telomerase activity [7,26]. Previous studies on *hTERT* promoter have defined a core region encompassing 330 bp upstream of the translation start site to 228 bp downstream, extending right into the second exon of the gene [28,29,30]. A number of transcription factor binding sites have been identified in this core promoter. However, the molecular mechanism underlying *hTERT* gene activation during induced Pluripotent Stem (iPS) cell reprogramming [31,32] and *hTERT* gene silencing during cellular differentiation remains largely unclear. On the other hand, recent studies have revealed the potential role of promoter mutations and chromosomal rearrangements in the activation of telomerase in cancer cells. These results have provided potential new strategies in targeting telomerase for cancer therapy. Here, we summarize the recent advances in the understanding of the transcriptional regulation of *hTERT* gene, focusing our attention on trans-acting factors, namely transcription factors and epigenetic modifiers, as well as genetic alterations in *hTERT* proximal region.

## 2. Trans-Acting Regulators of *hTERT* Transcription

**Figure 1:**
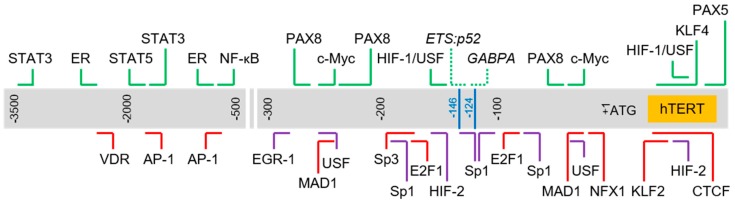
Schematic of transcription factor binding sites in human Telomerase Reverse Transcriptase (*hTERT*) promoter. Chromosomal sequence extending from 3.5 kb upstream and 150 bp downstream of *hTERT* translation start site (+1) is represented by the gray box. Horizontal lines above and below the box indicate approximate binding sites of respective transcription factors. Blue lines: hotspot promoter mutations (“-124” corresponds to C228T mutation; “-146” corresponds to C250T mutation); green: activator; red: repressor; purple: regulator with dual roles; dotted line: regulator bound to sites created by hotspot mutations.

The core promoter of the *hTERT* gene contains several known regulatory elements including GC-motifs and E-boxes. Several other articles have elegantly reviewed the roles specific factors or protein families play in the modulation of *hTERT* gene expression. Here, we have chosen to focus only on factors which have been reported to bind directly to the *hTERT* promoter region via in vitro or in vivo DNA–protein interaction assays, such as chromatin immunoprecipitation (ChIP) and electrophoretic mobility shift assay (EMSA) (refer to Table 1 for the complete list of factors). We selected a number of well-studied factors in each category and briefly discuss its role in the regulation of the *hTERT* gene, specifically highlighting the complexity of the regulatory network involved in controlling the expression of *hTERT*. As expected of a critical gene, *hTER*T transcription is modulated in a context-dependent manner via a multi-tiered regulatory network involving, among other means, feedback loops, and genetic and epigenetic controls. We also highlight the complexity of the *hTERT* proximal promoter with regards to the numerous response elements enclosed in this region (refer to Figure 1 for a schematic of the binding sites of selected transcription factors found in this region).

### 2.1. Transcription Activators of hTERT

#### 2.1.1. c-Myc

c-Myc, together with its dimerization partner Max, binds to regulatory elements called E-boxes and recruits histone acetyltransferases (HATs) to exact an activating effect on the transcription of various genes. Human *TERT* gene is one of them; c-Myc binds to two E-box sequences found on the core promoter of *hTERT*, leading to the upregulation of the expression of the gene and telomerase activity [43,44,45]. Mutating these sites weakens the promoter activity of *hTERT* gene [45,46]. In addition, overexpression of c-Myc in squamous cell carcinoma cells and human foreskin keratinocyte cells resulted in the upregulation of the *hTERT* promoter activity [46]. The transcription activating role of c-Myc on *hTERT* gene is mediated by the recruitment of the histone acetyltransferase (HAT) complex called SPT3-TAF9-GCN5 acetyltransferase complex (STAGA) and the transcription co-activator Mediator complex [66].

On the other hand, c-Myc alone may not be sufficient to drive the activation of *hTERT* expression. E6-transduced human foreskin keratinocytes (HFKs) did not show an increase in c-Myc expression, even though the cells attained replicative immortality [205]. This suggests that additional factors may be required in order to upregulate *hTERT* expression in these cells. Indeed, c-Myc was found to act cooperatively with Specificity Protein 1 (Sp1) in the activation of *hTERT* transcription via combinatorial binding of these two factors on their respective cis elements in the *hTERT* promoter [53]. When the E-boxes and GC-rich motifs (response elements of Sp1) were mutated, E6-mediated activation of telomerase expression was abolished. This also explains the observation that c-Myc and Sp1 expression correlates with *hTERT* transcription in various cancer cell lines.

Besides Sp1, numerous other factors play a role in modulating c-Myc-mediated regulation of *hTERT* transcription. Estrogen has been shown to activate c-Myc expression in breast cancer cell line, MCF-7 [51]. This, on top of direct activation of *hTERT* by estrogen receptor (ER) (see below), enhances *hTERT* transcription and telomerase activity in these cells. Aurora-A activates c-Myc expression and thence *hTERT* promoter activity in ovarian and breast epithelial cancer cells [61]. Nuclear Transcription Factor, X Box-Binding Protein 1 variant 123 (NFX1-123) and c-Myc, together with an unknown factor which acts upstream of the *hTERT* core promoter, co-activate *hTERT* gene expression in human foreskin keratinocytes [48]. Survivin induces the phosphorylation of c-Myc (and Sp1) and enhances its transactivation of *hTERT* transcription [63]. p300 interacts with and stabilises c-Myc via the latter’s Topologically Associated Domain (TAD) and they both co-activate *hTERT* gene expression [49]. Protein Kinase C θ (PKCθ) activates Nuclear Factor κB (NF-κB) signalling which in turn enhances c-Myc binding to *hTERT* promoter in activated human T lymphocytes [64]. ETS Proto-Oncogene 2 (Ets2) interacts with c-Myc and together they bind to *hTERT* promoter sequence and mediate breast cancer proliferation [52]. Colony Stimulating Factor 1 Receptor (CSF1R), upon induction by its ligand Colony Stimulating Factor 1 (CSF1), gets internalised into activated immortalized epithelial cells which lead to elevated binding of c-Myc to *hTERT* promoter [68]. Protein deglycase DJ-1 regulates c-Myc expression and thus *hTERT* promoter activity in renal and ovarian carcinoma cells [70]. Leptin upregulates binding of c-Myc (and Signal Transducer and Activator of Transcription 3 (STAT3)) to *hTERT* promoter and induces *hTERT* expression in HepG2 cells [71]. Brain-Derived Neurotrophic Factor (BDNF) activates *hTERT* expression and telomerase activity in spinal cord motor neurons via Mitogen-Activated Protein Kinase (MAPK)/Phosphatidylinositol-4,5-Bisphosphate 3-Kinase (PI3K) pathway which induces, among others, c-Myc expression [72]. Matrix Metallopeptidase 9 (MMP-9) facilitates in the switch from repressive Mad/Max-bound state to activating c-Myc/Max-bound state of the *hTERT* promoter in glioma cells [60]. Sirtuin 1 (SIRT1) upregulates c-Myc expression through activation of Forkhead Box O3A (FOXO3a) in human umbilical cord fibroblast (HUC-F2) cells and this leads to increased *hTERT* expression and longer lifespan of the cells [73]. The tumor suppressor p53 and its family members, p63 and p73, are potent repressors of *hTERT* transcription as transient overexpression of these factors in human embryonic kidney cells results in lower c-Myc expression and consequently attenuated *hTERT* promoter activity [88].

Conversely, the activity of the positive regulators of c-Myc-induced *hTERT* activation may be counteracted by a variety of c-Myc inhibitors and suppressors. A major player is Mad, a potent antagonist of c-Myc, whose mechanism of action involves competing with c-Myc for E-box protein binding sites found in transcription regulatory regions of their target genes (see below for the discussion on the role Mad plays in repressing *hTERT*). In addition, the lipid ceramide was shown to destabilize c-Myc protein via ubiquitination of the transcription factor, resulting in the downregulation of *hTERT* expression [45]. Other factors were shown to disrupt c-Myc binding to *hTERT* promoter and its transcription activation: Breast cancer 1 (BRCA1) associates with c-Myc via its N-terminal domain and depletes *hTERT* promoter-bound c-Myc in ovarian, prostate and breast cancer cells [81,82]. Cyclin-Dependent Kinase Inhibitor P27 (p27KIP1) interferes with c-Myc binding to *hTERT* promoter in malignant glioma cells [206]. Hypoxia-Inducible Factor 1-alpha (HIF-1α) downregulates c-Myc-mediated activation of *hTERT* promoter activity in colorectal carcinoma cells [77]. Mothers Against Decapentaplegic Homolog 3 (Smad3), upon Transforming Growth Factor beta (TGFβ) induction, interacts with c-Myc which leads to the downregulation of *hTERT* promoter activity [79,80]. TGFβ has also been shown to inhibit *hTERT* expression by upregulating Snail expression in human embryonic kidney (HEK) cells and keratinocyte cells [80]. Wilms Tumor 1 (WT1) downregulates *hTERT* transcription in clear cell renal cell carcinoma (ccRCC) by directly binding to *hTERT* promoter or by repressing c-Myc expression via its promoter activity [35]. Gastrokine 1 (GKN1) was reported to inhibit *hTERT* expression in gastric cancer cells by binding directly to c-Myc and downregulating its expression, leading to lower *hTERT* promoter activity [207]. The compound arsenic trioxide (ATO) was previously shown to downregulate telomerase activity and this was recently shown to involve the downregulation of four transcription activators of *hTERT*, one of which is c-Myc [101]. Knockdown of c-Myc (or the other factors) via siRNA could sensitize promyelocytic leukemia cells to ATO-induced apoptosis and inhibition of cell growth.

Despite the strong evidences brought forth by the various studies above regarding the key role c-Myc plays in the modulation of *hTERT* expression and telomerase activity, several studies involving the use of primary tumor samples have proven the lack of correlation between c-Myc expression and *hTERT* mRNA levels, specifically in hepatocellular carcinoma and breast carcinoma tissue samples [208,209]. Consistent with this notion, while c-Myc is one of the four transcription factors used for iPS cell reprogramming, it is non-essential [210].

#### 2.1.2. NF-κB

NF-κB is a transcription factor complex whose activity is induced in many cell types by various stimuli such as inflammation, cellular differentiation, tumorigenesis, and apoptosis. It is shown to play an activating role in telomerase expression and activity by regulating *hTERT* gene transcription via binding to the proximal promoter of the target gene, or indirectly by modulating the expression of transcription factors known to affect *hTERT* expression. The activation of NF-κB in primary bovine aortic endothelial cells (BAECs) and neuroepithelioma cell line via exposure to ionizing gamma radiation, and in human monocyte cells undergoing inflammation, leads to increased binding of NF-κB to *hTERT* promoter and consequently enhanced telomerase activity [147,148,152]. Depleting NF-κB levels by ectopically expressing NFKB Inhibitor Alpha (IκBα) or by disrupting the binding of NF-κB to *hTERT* promoter by eliminating its response element compromises the radiation- and lesion-induced upregulation of *hTERT* expression NF-κB-mediated activation of telomerase was also shown to be crucial in the recovery of intimal smooth muscle cells upon vascular injury in mediating intimal hyperplasia [57]. It was also proposed that there exists a feed-forward regulation between NF-κB and telomerase as the latter was found to bind to p65, a component of NF-κB, and modulate its transcription activity on its target genes, including factors which are important for inflammation and cancer progression [211,212]. The NF-κB response element in *hTERT* promoter is located more than 600 bp upstream of the translation start site, however, recently, the non-canonical NF-κB pathway was implicated in tumorigenesis specifically via a hotspot *hTERT* promoter mutation—C250T—which creates a binding motif for E-twenty-six (ETS) protein, a transcription activator of *hTERT* gene. Binding of ETS to the newly formed response element is not enough to activate *hTERT* transcription; it requires an activated non-canonical NF-κB signaling background to drive this transcription [213]. The NF-κB subunit, p52, is recruited to the C250T site and binds to its own half site and this facilitates the stimulation of *hTERT* transcription in the cancer cells.

As mentioned, NF-κB can also activate *hTERT* expression via an indirect way. It is known to activate strong *hTERT* transcription activators such as c-Myc and Sp1 in a number of human cell lines [58,64]. Besides the ones described above, other naturally occurring and synthetic chemicals were shown to modulate NF-κB-mediated activation of *hTERT* transcription. The plant-derived molecule, curcumin, and the drug, pelitinib, were both shown to potently inhibit NF-κB-induced *hTERT* activation by ionizing radiation in neurogenic cancer and tongue squamous cell carcinoma cells, respectively [148,153]. In addition, arsenic trioxide (ATO) was found to repress the expression of NF-κB (and other transcription factors namely Sp1, c-Myc and Upstream Transcription Factor 2 (USF2)) in human promyelocytic leukemia cells and reduce the transcription of *hTERT* [101].

#### 2.1.3. STAT Proteins

STAT3 plays a key regulatory role in the expression of *hTERT* in several cancer cell lines including gastric, breast and glioblastoma, and *hTERT* in turn contributes to the survival of these STAT3-dependent tumors. When STAT3 expression levels were reduced in a hepatocellular carcinoma (HCC) cell line using siRNA, *hTERT* expression was consequently reduced [214]. Studies involving breast cancer stem cells revealed that STAT3 binds to and activates *hTERT* promoter in concert with Cluster of Differentiation 44 (CD44) and NF-κB, and that diminished levels of STAT3 resulted in the downregulation of *hTERT* expression [149]. Mechanistically, STAT3-mediated upregulation of *hTERT* expression may be the result of the following biological factors: leptin induction in breast cancer and HCC cells [71,188], miR-21 expression in glioblastoma cells [191], and core protein of hepatitis C virus (HCVc) in HCC cells [192]. STAT3-mediated upregulation of *hTERT* expression by HCVc also involves the action of DNA Methyltransferase 1 (DNMT1) which facilitates the methylation of *hTERT* promoter.

STAT5 has been shown to bind directly to a distal promoter region of *hTERT* in a chronic myelogenous leukaemia (CML) cell line and an adult T-cell leukaemia (ATL) cell line, resulting in the activation of telomerase expression [193,194]. The positive effect STAT5 has on *hTERT* expression was also seen in primary leukemic cells upon Interleukin 2 (IL-2) induction and has been attributed to the phosphorylation of STAT5 which permits its interaction with Janus-activated kinase (JAK)-1 and -2. Depletion of STAT5 via the introduction of specific siRNA was shown to lead to the inhibition of IL-2-induced *hTERT* activation [193]. However, STAT5 was also reported to indirectly activate *hTERT* through c-Myc, another transcription activator of *hTERT*. In two different leukemic cell lines, erythropoietin (EPO) was proven to be a potent activator of telomerase and this was shown to be via the induction of the JAK2/STAT5/c-Myc axis, and in turn was negatively regulated by TGFβ/Smad3 signaling [195,196].

#### 2.1.4. Paired Box Proteins (Pax)

Pax protein family consists of paired box- and homeobox-containing transcription factors which play a crucial role in early development. Two of its members were shown to bind to regions proximal to *hTERT* translational start site and activate its transcription. In lymphocyte cell lines, PAX5 was found to bind to two sites, one each in exon 1 and 2 of *hTERT* gene [156]. Knockdown via siRNA and overexpression of PAX5 lead to increased and decreased *hTERT* expression, respectively. Furthermore, these effects were reported to be independent of CCCTC-binding factor (CTCF) and promoter methylation. On the other hand, PAX8 was revealed to bind to four sites upstream of the *hTERT* translational start site in glioma cells [157]. Interestingly, PAX8 was also shown to bind and activate hTR promoter, which fortifies the role of PAX8 in the regulation of telomerase activity.

#### 2.1.5. Estrogen Receptor 

Estrogen receptor is a nuclear hormone receptor which binds to estrogen response elements (ERE) upon stimulation by its ligand. *hTERT* transcription and telomerase activity are found to be activated by estradiol (E2) in ER-positive cells but not in ER-negative ones [215]. However, artificial expression of ER in ER-negative cells was found to result in *hTERT* transcription activation and increased telomerase activity. In vitro DNA-protein binding assays revealed that ERα specifically binds to two EREs in the *hTERT* promoter [51,116,118]. In addition, several biomolecules were shown to inhibit ER activation of *hTERT* in human cancer cells. Treatment of breast cancer cells with indole-3-carbonol (I3C) [121], colon cancer cells with 15-deoxy-Δ12,14-prostaglandin J2 (15d-PGJ2) [84], ovarian cancer cells with miR-498 [120], and breast and endometrial cancer cells with progesterone [122], were reported to result in the suppression of ER-induced activation of *hTERT* expression.

### 2.2. Transcription Repressors of hTERT

#### 2.2.1. Mad1

Mad1 is an antagonist of Myc protein in that it competes with the latter for E-box motif binding in promoter region of target genes and mediates the repression (as opposed to Myc’s activation) of these genes. The binding of Mad1 to *hTERT* proximal promoter is mediated by an N-terminal domain which is responsible for its interaction with its co-repressor, Sin3A [139]. This binding can be reversed by overexpression of Myc in mortal WI38 cells [86]. The Mad1-induced *hTERT* repression has clinical significance in patients suffering from myelodysplastic syndrome (MDS) as these individuals, especially those with more favorable outcomes, show higher expression levels of Mad1 as compared to healthy controls [216]. The authors of the report went on to propose that Mad1 plays an important role in reducing *hTERT* expression during the early stage of the disease. Thus, it is not surprising that a number of publications have focused on the effects of various molecules on Mad1-mediated repression of *hTERT* promoter. For example, 4-[3-(1-adamanyly)-4-hydroxyphenyl]-3-chlorocinnamic acid (3-C1-AHPC), an adamantyl-related molecule, has been shown to inhibit the expression of miR-202, which targets Mad1 gene, and hence lead to de-repression of *hTERT* gene and increased telomerase activity [140]. The repressive role of the 3-C1-AHPC/miR-202/Mad1 axis on *hTERT* expression was supported by the observation that the overexpression of pre-miR-202 and inhibition of miR-202 resulted in the decreased and increased repressive activity of Mad1 on *hTERT* expression, respectively. In addition, EGCG (a green tea polyphenol), sulforaphane (SFN), 15d-PGJ2 (15-deoxy-delta-12,14-prostaglandin J2), selenium, and 12-*O*-tetradecanoylphorbol-13-acetate have been shown to enhance Mad1’s binding to and repression of *hTERT* promoter [85,108,139,217,218].

#### 2.2.2. Sp3

Sp3 is a GC-box-binding protein with an antagonistic function to Sp1. Sp3, together with Sp1, has been shown to bind to a recognition site in the proximal promoter of *hTERT*, resulting in the repression of the gene. This binding is important but not sufficient to govern *hTERT* expression; chromatin environment, such as histone modification patterns, is also essential [219]. In fact, several studies have reported that the binding of Sp3 to *hTERT* proximal promoter leads to the recruitment of a histone deacetylase (HDAC), HDAC1, to the promoter region and, in turn, modifies the epigenetic landscape of the region, resulting in the silencing of the *hTERT* gene [186]. This repression can be reversed by overexpression of dominant-negative form of Sp3. On the other hand, ceramide is known to enhance the Sp3/HDAC-mediated suppression of the *hTERT* gene [174]. This involves the ceramide-induced deacetylation of Sp3, an active form of the protein with higher binding capacity to *hTERT* promoter, and a decrease in RNA polymerase II recruitment to the promoter due to lower histone acetylation levels in the region. Ceramide has also been shown to reduce Sp1 binding to the *hTERT* promoter, thus abrogating its activating effect on *hTERT* transcription [174]. However, it is worth noting that a study has posited that Sp1, but not Sp3, plays a regulatory role in telomerase activity in Jurkat T cells, as Sp3 overexpression in these cells did not cause a significant change in *hTERT* mRNA levels or telomerase activity [220].

#### 2.2.3. CTCF

CTCF is chromatin-binding factor which affects transcription of numerous genes. It mostly acts as a transcription repressor but is known to activate the transcription of several genes. The binding of CTCF to the first exon of the *hTERT* gene was reported to suppress its expression in telomerase-negative cells but not in cancer cells [105]. It preferentially binds to unmethylated sites of the *hTERT* regulatory region. The treatment of telomerase-positive cells with a strong demethylating agent (5-azadC) led to the reactivation of CTCF binding to its response element and repression of *hTERT* expression. This observation was supported by another study whose results showed that the introduction of sulforaphane (SFN), a potent histone deacetylase inhibitor, in human breast cancer cells resulted in CpG demethylation and hyperacetylation of the region proximal to the first exon of *hTERT* and this facilitated the binding of *hTERT* repressors, such as MAD1 and CTCF, in this region [108]. Conversely, siRNA-induced knockdown of CTCF was sufficient to partially reverse the inhibitory effect of SFN on *hTERT* transcription. In addition to binding to the first exon of *hTERT*, CTCF was also found to negatively modulate *hTERT* expression by binding to an enhancer element approximately 4.5 kb upstream of the *hTERT* transcription start site and this was confirmed by chromosome conformation capture (3C) assay [221].

#### 2.2.4. E2F1

E2F1 is a transcription factor which binds to E2 recognition sites found in the promoter of numerous genes, especially those involved in cell cycle regulation and DNA damage response. It has also been shown to play a direct role in the transcription regulation of *hTERT* in human squamous cell carcinoma cells by binding to and modulating *hTERT* proximal promoter [110]. The overexpression of wildtype E2F1, but not its mutant, has been shown to downregulate *hTERT* promoter activity and telomerase activity. The effect of E2F1 on *hTERT* promoter has been proposed to counteract the activating effect of c-Myc on the regulatory region [100] and act downstream of the TGFβ signaling pathway as the expression of a dominant negative form of E2F1 resulted in the abrogation of the TGFβ-induced *hTERT* inhibition [111].

#### 2.2.5. Hormone Nuclear Receptors

Vitamin D(3) receptor (VDR) is the nuclear receptor for 1,25-dihydroxyvitamin D(3) (VD3) and it can form a heterodimer with another nuclear receptor, retinoid X receptor (RXR), whose ligand is 9-cis-retinoic acid (9-cis-RA). In prostate cancer cells, this heterodimer was found to bind directly to a region approximately 2.5 kb upstream of *hTERT* translation start site and repress the expression of the gene [203]. In vivo experiments involving xenografts in nude mice recapitulated the inhibitory effects of this protein heterodimer on *hTERT* transcription. However, another report has argued against the direct role of VDR on *hTERT* transcription repression. Instead, it was proposed that the inhibitory effect of VD3 stems from its suppression of *hTERT* mRNA by destabilizing it [222]. This was supported by independent studies in ovarian, endometrial and breast cancer cells where VD3 was shown to repress *hTERT* expression through the induction of miR-498, which in turn targets the 3’ untranslated region (3’ UTR) of *hTERT* transcripts and thus decreases its expression [120,223].

Another nuclear receptor that has a repressive effect on *hTERT* transcription is androgen receptor (AR). Treatment of prostate cancer cells with AR agonist resulted in the inhibition of *hTERT* promoter activity and, conversely, treatment of AR antagonist failed to recapitulate this inhibition [33]. On top of that, the expression of a mutant form of AR (T877A) not only broadens ligand specificity but also precluded its binding to the *hTERT* promoter.

### 2.3. Transcription Regulators with Dual Roles

#### 2.3.1. Specificity Protein 1 (Sp1)

Sp1 is a transcription factor that binds to GC-box motifs in the promoter of its target gene and regulates its expression, either activating or repressing the transcription process depending on cellular context. Sp1 is known to activate *hTERT* gene expression in telomerase-positive cells but suppresses it in telomerase-negative ones. It does this by binding to five GC-box motifs found in the proximal promoter of *hTERT* [46,165]. Mutation of these binding sites results in the attenuation of Sp1-mediated *hTERT* activation or repression in various cell lines. However, the expression and *hTERT* promoter-binding of Sp1 alone is insufficient to drive the desired effect. Epigenetic environment, especially the presence of suitable histone marks, is crucial in actualising these effects.

In telomerase-positive cells, particularly cancer cells, Sp1 may activate *hTERT* expression on its own or in conjunction with specific co-activators. Sp1 may work cooperatively with c-Myc and bind their respective response elements in *hTERT* proximal promoter to upregulate the transcription of the gene [46,53]. In fact, in the various cell lines examined, expression of Sp1 and c-Myc correlates positively with *hTERT* transcription activity. Kaposi’s sarcoma-associated herpesvirus (KSHV) latent protein, latency-associated nuclear antigen (LANA), was also found to bind directly to Sp1 in KSHV-infected body-cavity-based lymphoma (BCBL) cells and enhance the binding of Sp1 to its cognate binding sites in the *hTERT* proximal promoter [165,166]. In HeLa cells, the human T cell leukaemia virus type 1 protein, HTLV-1 bZIP factor (HBZ), forms heterodimers with JunD and this complex interacts with Sp1 at the *hTERT* promoter region and causes the activation of the target gene [36]. In addition, MBD1-Containing Chromatin-Associated Factor 1 (MCAF1) was shown to interact with Sp1 and the general transcription machinery in HeLa cells and facilitate *hTERT* expression [168]. Nuclear Factor Of Activated T-Cells 1 (NFAT1) can bind to *hTERT* proximal promoter at a site flanked by two GC-boxes and this allows for the synergistic interaction between NFAT1 and Sp1, resulting in the activation of *hTERT* transcription [154]. In human liver carcinoma cells, High Mobility Group AT-Hook 2 (HMGA2) binds to Sp1 and disrupts the recruitment of histone deacetylase (HDAC) to the promoter of *hTERT* and this leads to an increase in the expression of the gene [170]. This observation was recapitulated in cells treated with suberoylanilide hydroxamide (SAHA), a HDAC inhibitor. Numerous other factors and molecules have been shown to facilitate Sp1-mediated upregulation of *hTERT* gene expression in various cell lines (refer to Table 1 for a comprehensive list), and many others have been reported to play a repressive role instead. Notable inhibitors of Sp1-mediated activation of *hTERT* promoter activity include the tumor suppressor p53 [171,224] and its family members p63 and p73 [88,172,173], the key transcription factor E2F-1 [176 Beitzinger, 2006 #55], and the cell cycle checkpoint proteins p27Kip1 [93] and p16 [225].

As alluded to earlier, epigenetic environment is important in Sp1-mediated regulation of *hTERT* gene. The epigenetic mechanism behind the repression of *hTERT* gene expression in telomerase-negative human somatic cells is particularly well-characterized. Sp1 binds to its cognate binding motifs found in the *hTERT* proximal promoter and recruits HDAC proteins to this region [183,226]. The deacetylation of histone subunits leads to the silencing of the *hTERT* gene. This transcription repression may be reversed by treating the cells with trichostatin A (TSA), a HDAC inhibitor [226,227]. TSA-induced activation of *hTERT* was shown to be enhanced by the overexpression of Sp1 [227] or HDAC1 [226], or the mutation of the Sp1 binding sites [226,227]. In contrast, TGF-β-activated kinase 1 (TAK1) has the ability to facilitate the recruitment of HDAC to Sp1 on *hTERT* promoter and suppress *hTERT* activation in human lung adenocarcinoma cells [185].

#### 2.3.2. Activator Protein 1 (AP-1)

AP-1 is a transcription factor complex which consists of components belonging to the c-Jun, c-Fos, Activating Transcription Factor (ATF) and J Domain-Containing Protein (JDP) families. Its effect on *hTERT* transcription has been contentious. It was initially reported to repress *hTERT* expression in HeLa cells, where the transient overexpression of its components (c-Fos and c-Jun or c-Fos and JunD) strongly represses *hTERT* promoter activity [34]. This suppression was found to be mediated by the binding of the heterodimer to two regions upstream of the *hTERT* translation start site. Mutation of these binding sites led to the abrogation of the repressive effect of AP-1 on *hTERT*. On the other hand, ectopic expression of the viral protein HBZ and JunD in HeLa cells was shown to activate *hTERT* promoter [36]. This involves the interaction of HBZ/JunD heterodimer with the co-activator Sp1 and their binding to GC-rich motifs in the *hTERT* promoter.

#### 2.3.3. Early growth response-1 (EGR-1)

EGR-1 is a transcription factor whose activity is crucial for mitogenesis and cellular differentiation. The effect of this protein on *hTERT* transcription is two-fold—it can act as an activator and also a repressor depending on the tissue of origin. Ectopic expression of EGR-1 in villous cancer cells led to an increase in *hTERT* expression, both in the mRNA and protein levels [114]. This effect was shown to be mediated by the direct binding of the protein to a single site in the proximal promoter region of *hTERT*. Furthermore, expression of EGR-1 was found to correlate with that of *hTERT* during trophoblastic development and in patients with preeclampsia. On the contrary, the examination of mRNA levels in primary cervical cancer tissues revealed a negative correlation between the expression of EGR-1 and that of *hTERT*. Further studies involving epidermoid carcinoma and squamous cell carcinoma cell lines supported this observation—overexpression of EGR-1 resulted in the suppression of *hTERT* expression [115]. These results showcased the important role EGR-1 plays in the regulation of *hTERT* gene transcription.

#### 2.3.4. Hypoxia-inducible factor 2-alpha (HIF-2α)

HIF-2α is a transcription factor which is involved in cellular oxygen regulation. Unlike another hypoxia-inducible factor, HIF-1, whose regulatory effect on *hTERT* gene is solely positive, HIF-2α plays dual role in modulating *hTERT* expression. In several renal carcinoma cell lines tested, HIF-2α overexpression led to an increase in *hTERT* promoter activity and its depletion resulted in lower *hTERT* mRNA levels [134]. This positive effect is mediated by the direct binding of HIF-2α to *hTERT* proximal promoter and the recruitment of p300 and histone acetyltransferase to the regulatory site. In contrast, ectopic expression of HIF-2α in three glioma cell lines led to repression of *hTERT* gene expression [134]. This suggests that HIF-2α may play different roles in *hTERT* gene regulation in different cellular context.

#### 2.3.5. Kruppel-like family of transcription factors (KLF) Proteins

KLFs are a group of proteins which share a common structure, namely a C-terminal domain consisting of three zinc finger configurations. The human genome contains 17 KLF family genes and they may act as activators or repressors of their target genes. Two of the members of the KLF family proteins are known to play opposite roles in the regulation of *hTERT* expression. KLF2 was shown to bind to a putative site in the first exon of the *hTERT* gene in resting T cells, resulting in the repression of the latter [136]. However, upon activation of these cells, KLF2 dissociates from the regulatory element and this relieves the gene of its repression. In contrast, KLF4, a key pluripotency marker, and mediator, was found to activate *hTERT* expression in telomerase-negative, alternative lengthening of telomere (ALT) cells and human fibroblast cells via direct binding to a response element in the proximal promoter region of *hTERT* [137]. Conversely, knocking down KLF4 in telomerase-positive cancer and stem cells abrogated *hTERT* expression significantly. Furthermore, telomerase expression was found to be sufficient in replacing KLF4 function in the maintenance of self-renewal in human embryonic stem cells, showcasing that *hTERT* is one of the primary targets of KLF4 in stem cell maintenance. Interestingly, a recent study revealed that the knockdown of *hTERT* via shRNA in HeLa cells led to a decrease in KLF4 expression [228]. This shows that the interplay between the expression of KLF4 and *hTERT* is a complex and important one.

#### 2.3.6. Nuclear Transcription Factor X Box-Binding Protein 1 (NFX1)

NFX1 is traditionally known as a transcription repressor. It plays a key role in determining the duration of inflammatory response via its interaction with and transcription regulation of the promoter of MHC class II genes. Three isoforms of NFX1 have been identified and two of these have opposite roles in the regulation of *hTERT* gene expression. NFX1-123, with c-Myc as a co-activator, was shown to bind to an X-box motif located adjacent to a known E-box motif, the canonical response element of c-Myc and other E-box proteins, and activate *hTERT* promoter activity [48]. In contrast, NFX1-91 represses *hTERT* expression by binding to the same X-box motif and recruiting Sin3A and histone deacetylases (HDACs) to the proximal promoter of *hTERT* [48]. This repressive effect can be reversed by the expression of the human papillomavirus (HPV) viral protein E6, which facilitates the ubiquitination and degradation of the NFX1-91 isoform, or by knocking down NFX1-91. Thus, alternative splicing of the NFX1 transcript plays an important role in the expression of *hTERT* gene in human fetal kidney cells.

#### 2.3.7. Upstream stimulatory factors (USF) Proteins

USFs are a group of proteins belonging to the basic helix-loop-helix leucine zipper protein family. They function as transcription regulators and are known to bind to E-box motifs found in regulatory elements of their target genes. USF1 and USF2 have been shown to play both activating and repressive roles in the regulation of *hTERT* gene expression. USF1/2 heterodimers were found to bind directly to E-box motifs found on the proximal promoter of *hTERT* in both telomerase-positive and -negative cells. However, they specifically activate *hTERT* expression only in telomerase-positive cells [200]. This positive effect was shown to be mediated by p300, which acts as a co-activator, and p38 MAP kinase, a known activator of USF activity. In stark contrast, two other reports posited that USF1 and USF2 act as suppressors of *hTERT* expression in telomerase-positive oral squamous cancer cells and E6-expressing immortalized cells [54,202]. This was evident by the downregulation and upregulation of E6-mediated *hTERT* expression upon the forced expression and siRNA-induced knockdown of the USF genes, respectively. Moreover, binding of USF1 and USF2 to the proximal E-box motif in the *hTERT* promoter diminishes in cells expressing E6 and this is accompanied by an increase in c-Myc binding to the same site [54]. Interestingly, yet another group proposed that the effect USF2 has on *hTERT* expression is dependent on the relative abundance of the splice isoforms in the cells. They showed that both full-length and truncated forms of USF2 can bind to the *hTERT* proximal E-box. However, the latter has a dominant-negative effect on the former, therefore depleting the full-length isoform and abrogating its activating effect on *hTERT* expression [201]. This concurs with the finding that the N-terminally truncated isoform is only present in telomerase-negative resting lymphocytes but not in telomerase-positive activated lymphocytes [201].

### 2.4. Epigenetic Modifiers Regulating hTERT Transcription

Differentiation of pluripotent stem cells is accompanied by the downregulation of *hTERT* expression. *hTERT* promoter is eventually silenced when the cells are terminally differentiated. On the other hand, reprogramming of somatic cells to induced pluripotent stem cells results in the activation of *hTERT* expression. The epigenetic regulation of *hTERT* promoter plays an important role in these two opposite *hTERT* transcriptional states. The following section will focus on the known modes of epigenetic regulation of *hTERT* expression including methylation of *hTERT* promoter, histone modifications, and modulation by sirtuins and non-coding RNAs. However, it remains unclear how *hTERT* expression is differentially regulated during cancer development, cellular differentiation and reprogramming.

#### 2.4.1. Histone Modifiers

In telomerase-positive cells, telomerase expression is associated with hyperacetylation of histone H3 and H4 and methylation of lysine-4 of histone H3, whereas in telomerase-negative cells (ALT cells), the silencing of the expression of telomerase is associated with hypoacetylation of H3 and H4 and methylation of lysine-9 of histone H3 [229]. Induction of the tumor suppressor, AT-Rich Interaction Domain 1A (ARID1A), increases occupancy of H3K9me3 at transcription start site (TSS) of *hTERT* and decreases acetylated lysine-12 of histone H4 (H4K12Ac) levels at this site. Treatment of telomerase negative cells with histone deacetylase inhibitor (HDACi), trichostatin A (TSA), could activate transcription of *hTERT* gene [229].TSA was shown to inhibit deacetylation of histone H3 lysine-9 or -14, leading to upregulated *hTERT* expression in umbilical cord mesenchymal stem cells as well [230]. Sirtuin protein family consists of seven homologs of yeast Sir2 protein which is known to be involved in a plethora of biological processes including aging [231], stress response, and tumorigenesis [232]. SIRT1, also known as nicotinamide adenine dinucleotide (NAD)-dependent deacetylase sirtuin-1, was shown to deacetylate c-Myc [233]. Deacetylated c-Myc displays enhanced association with Max, an essential partner for its activation [227], and this, in turn activates *hTERT* expression [233]. Knocking down SIRT1 leads to decreased expression of genes targeted by c-Myc including *hTERT*. These findings have been recapitulated by other groups with additional mechanism. Deletion of SIRT1 leads to significant reduction in telomerase expression, thus causing telomere dysfunction-induced foci and nuclear abnormality [234,235]. The same group found that deletion of SIRT1 is associated with substantial induction of acetylated lysine-9 of histone H3 (H3K9Ac) and reduction in trimethylated H3K9 at *hTERT* promoter [235]. In addition, SET and MYND Domain-Containing Protein 3 (SMYD3) knockdown in colorectal carcinoma and hepatocellular carcinoma cells was found to diminish the occupancy of c-Myc at *hTERT* promoter via reduction in H3K4 trimethylation and histone H3 acetylation.

#### 2.4.2. Regulators of *hTERT* Promoter Methylation

DNA methylation is involved in gene silencing [236,237]. The *hTERT* promoter region contains clusters of CpG islands with dense GC-rich regions. This suggests that DNA methylation may play a role in the regulation of *hTERT* expression [238]. However, recent reports suggested that there is little or no methylation around the *hTERT* transcription start site (TSS) in most cancer cell lines and embryonic stem cells [239,240]. Consistent with these results, demethylation of CpG by demethylating agent 5aza-2′-deoxycytidine (5azadC) is not associated with increased *hTERT* expression in cancer cells [105,227,241,242]. In summary, there is an inconclusive correlation between DNA methylation and telomerase regulation. Information on the detailed results for different tissues, and regions of *hTERT* promoter, data interpretation, and categorization, and the DNA methylation detection methods is summarized in Table 2.

### 2.5. Monoallelic Expression of hTERT Gene

Several cancer cell lines contain point mutations in only one of its *hTERT* alleles, specifically in its promoter region (see below for a detailed discussion of *hTERT* promoter mutations and their effect on *hTERT* expression). This is attributed to the reversal of *hTERT* gene silencing on the one active allele while the other one remains silenced. The promoter of the active gene was found to be decorated by the permissive histone H3K4me2/3 mark, while the promoter of the silenced gene showed higher deposition of H3K27me3, a histone mark indicating transcriptionally repressed heterochromatin regions [247]. Concomitant to this epigenetic switch in the *hTERT* promoter region, the point mutation allows for the binding of the transcription factor GA Binding Protein Transcription Factor Alpha and -Beta 1(GABPA/B1) heterodimer to the newly created site, which in turn facilitates the recruitment of RNA Polymerase II to the promoter, resulting in the transcription of *hTERT* gene.

### 2.6. Complexity of Trans-Regulation of hTERT Gene Transcription

It is clear from the multitude of evidence presented above that *hTERT* transcription regulation is complex. It involves the interplay between the various components of molecular and cellular biology. This is not surprising given the crucial role telomerase plays in the maintenance of stem cell and cellular transformation. Despite the wealth of knowledge in the roles transcription factors and epigenetic modifiers play in the regulation of *hTERT* expression, a lot more has to be done to elucidate the mechanism underlying the switching off and on of *hTERT* gene during cellular differentiation and cellular reprogramming, and during cellular transformation, respectively.

## 3. Genetic Alterations Regulating *hTERT* Transcription

Recent advances in DNA sequencing technologies have enabled large-scale genome sequencing studies across various tumor types. Many alterations in protein-coding genes have been identified [248,249]. On the other hand, only a handful of the mutations in non-coding regions have been recently identified [250]. Recurrent mutations and chromosomal rearrangements in *hTERT* promoter have further confirmed the importance of telomerase activation in human cancers [251,252].

### 3.1. hTERT Promoter Mutations

Transcriptional regulation at the level of *hTERT* promoter mutations first came into attention with the discovery of highly recurrent mutations in this region in melanomas. In particular, two hotspot C > T point mutations were observed at the nucleotide position 124 bp and 146 bp upstream of the translation start codon (ATG) and these specific mutations were termed C228T and C250T, respectively. Other less common mutations were also detected in the *hTERT* promoter region such as the CC > TT mutations at −124/−125 and −138/−139 positions. The observation that the frequency of mutations detected in *hTERT* promoter is higher than those in the B-Raf Proto-Oncogene Serine/Threonine-Protein Kinase (*BRAF*) gene, and that the presence of highly recurrent point mutations in just two nucleotide positions strongly suggests that these are driver mutations which play key roles at various stages of tumorigenesis in melanoma [251,252].These important findings have provided new insights into a possible mechanism for *hTERT* activation in human cancers. In addition, the discovery of driver alterations in the non-coding portion of the human cancer genome was a novel advancement in understanding the role of mutated non-coding sequences in transcriptional deregulation and tumorigenesis.

#### 3.1.1. *hTERT* Promoter Mutations in Different Types of Human Cancers

**Figure 2:**
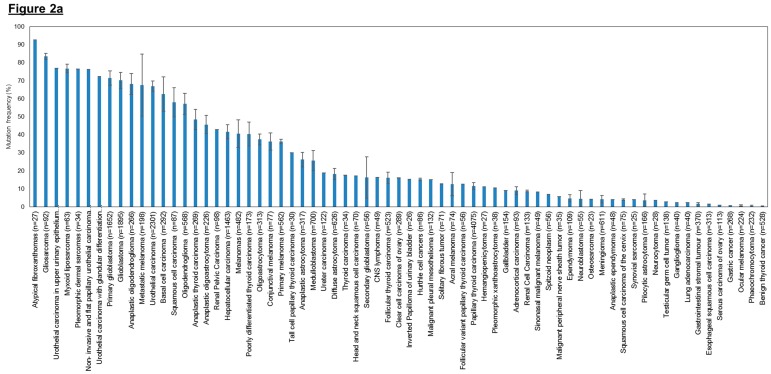

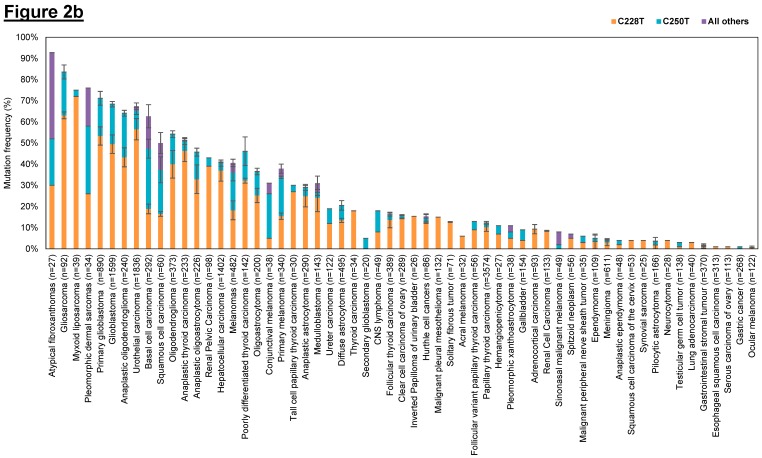
Frequency of human telomerase reverse transcriptase (*hTERT*) promoter mutations in various cancer types. (**a**) Overall *hTERT* promoter mutation frequencies of various cancer types plotted in descending order; (**b**) Overall *hTERT* promoter mutation frequencies of various cancer types with breakdown of individual frequencies of C228T, C250T, and all other mutations. The overall mutation frequencies of *hTERT* promoter were compiled from all relevant publications on human cancer genome sequencing. The label “*n*” corresponds to the total number of tumors sequenced among different studies for the same tumor type. Error bars correspond to the standard errors in mutation frequencies calculated among different studies on the same tumor type. Only studies with at least 20 samples sequenced were included in this study. Only studies which provided the detailed breakdown of different mutation sites were included in (**b**). Refer to Table 3 for the list of references used to compile this figure.

Since the publication of the studies mentioned above, there has been a surge in interest in investigating the frequency of *hTERT* promoter mutations in melanomas and other types of cancers, particularly at the two hotspot locations, C228T and C250T (Figure 2). To date, *hTERT* promoter mutations have been evaluated in more than 60 tumor types and they are found to be the most common point mutations in hepatocellular carcinoma [253,254], glioblastoma [253], bladder cancer [255] and melanoma [251,252].

Cancer types that have high overall mutation frequency generally correspond to tumors arising from cells with low turnover rate such as neurons, fibroblasts, glial cells and hepatocytes (Figure 2a). This observation concurs with previously published results that showed that *hTERT* promoter mutations occur most frequently in cancers that originate in tissues with low self-renewal rates [253]. Based on the *hTERT* promoter mutation studies conducted so far, it is tantalizing to hypothesize that cancer subtypes without *hTERT* promoter mutations may derive from tissues with tissue-specific stem cells as they already display high telomerase expression and therefore do not require *hTERT* promoter mutations to further upregulate telomerase expression to sustain telomere maintenance. In contrast, cells with lower *hTERT* expression levels and low turnover rates might selectively acquire *hTERT* promoter mutations during tumorigenesis to upregulate telomerase levels and avoid replicative senescence. We further examined the distribution of the two hotspot mutations (C228T and C250T) across the different tumor types (Figure 2b) and observed that the mutation frequency ratio of C228T:C250T is markedly lower in cancers of the cutaneous tissues such as basal cell carcinomas, squamous cell carcinoma and melanoma.

#### 3.1.2. Mechanism by Which *hTERT* Promoter Mutations Leads to Enhanced Telomerase Levels

The hotspot mutations mentioned earlier create a CCGGAA/T binding motif for ETS (E-twenty six) transcription factors whose recruitment to these sites may result in increased *hTERT* expression. This is facilitated by the activation of non-canonical NF-κB signaling pathway which provides a co-activator, in the form of p52, to enhance the transcription activity of the *hTERT* promoter [213] (see above for a more comprehensive discussion of the molecular mechanism). Reporter assays carried out to assess the functional impact of these promoter mutations showed a two- to four-fold increase in *hTERT* promoter activity in melanomas [251,252].

### 3.2. Chromosomal Rearrangements and hTERT Expression

A recent study involving neuroblastoma tumors posited the significance of genomic rearrangements in telomerase activation specifically in the high-risk bracket of this tumor type [246]. They discovered from their analysis of whole-genome sequencing data that neuroblastoma tumors displayed breakpoint clusters at four genomic loci and one of them is at the 5p15.33 region where *hTERT* gene is located. These chromosomal rearrangements were found to consistently cluster in the region about 50 kb upstream of the *hTERT* transcription initiation site, but at the same time leave the coding and core promoter region intact. They showed that these chromosomal alterations result in the juxtaposition of *hTERT* gene to strong enhancer regions, resulting in major changes in the epigenetic landscape of the affected region. These changes, in turn, lead to the abrogation of *hTERT* gene silencing engendered by repressive histone modification and DNA methylation states.

## 4. Conclusions

Herein, we have summarized the numerous transcription activators and repressors that were found to interact with the *hTERT* core promoter. However, it is still unclear how *hTERT* is silenced during stem cell differentiation as well as reactivated during somatic cell reprogramming. These processes are likely to involve a host of these transcription regulators in a cell context-dependent manner. In addition, they are likely to be controlled by epigenetic changes accompanying these cellular events. The contribution of specific transcription modulators in these processes remains to be explored and elucidated. Given the complexity of the regulatory network, it is perhaps more meaningful to approach the issue from a wider perspective by studying the network system as a whole instead of focusing on individual players.

The identification of *hTERT* promoter mutations, on the other hand, may provide interesting biomarkers for diagnosis and prognosis of various cancer types. Since the publication of the abovementioned landmark papers, many studies have subsequently explored the potential application of these mutations as biomarkers. To date, the existence of *hTERT* promoter mutations has been associated with decreased survival in patients suffering from melanoma [279,292], bladder cancer [280,352], urogenital cancer [280,325], glioma [274], medulloblastoma [316], thyroid cancer [299,300,330], and laryngeal tumors [353].

As shown in Figure 2, urothelial carcinoma samples display up to 67% mean mutation frequency (*n* = 2301) in the *hTERT* promoter region, specifically at the two hotspots, making it the most frequently mutated gene identified so far in this region. Hence, these *hTERT* promoter mutations have the potential to become a good biomarker for clinicians to conduct early detection of bladder cancer and subsequent follow-up tests for cancer progression or recurrence in patients. In hepatocellular carcinomas, about 42% mean mutation frequency (*n* = 1463) in the *hTERT* promoter region was observed. Similarly, their high prevalence suggests that these hotspot mutations may be utilized as candidate biomarkers for early detection and monitoring. Lastly, the high prevalence of *hTERT* promoter mutations in various subtypes of gliomas also suggest that the mutations can serve as useful biomarkers to aid in classification and prognostication via extraction of samples from cerebrospinal fluid. Further studies to confirm such a causal relation in various tumor types will be necessary before genome-based clinical classifications based on *hTERT* promoter mutations can find their way into the clinics.

Activation of telomerase enzyme is classified as one of the classic hallmarks of cancer as it confers cells with replicative immortality [354]. Hence, blocking the activity of telomerase is an active area of research in cancer therapeutics. Previous strategies to block telomerase activity in cancer patients have focused on the use of immunotherapy, gene therapy, small molecular inhibitors and G-quadruplex ligands, of which some have entered clinical trials [355]. However, such targeting strategies can also result in non-specific inhibition of telomerase activity in tissue progenitor/stem cells, which may limit the utilization of such telomerase inhibitors in the long run. As we find out more about the mechanism of how *hTERT* promoter is activated or repressed, novel strategies to therapeutically target telomerase can be developed. For instance, new specific small molecular inhibitors may be developed to interfere with the binding of ETS/TCF transcription factors to the CCGGAA/T binding motif that is only present in cancer cells that carry the specific C228T or C250T mutation. In addition, as mentioned above, the presence of *hTERT* promoter mutation in a tumor may be used as a biomarker to predict subsequent clinical response to a telomerase inhibitor drug.

## Figures and Tables

**Table 1:** List of factors reported to bind to human Telomerase Reverse Transcriptase (*hTERT*) promoter and regulate its expression.

Regulator	Activator/Repressor	Cell Tested	Binding Site	Binding Assay	Reference	Co-Regulator	Positive Regulators	Negative Regulators
Androgen receptor	Repressor	LNCaP, PC3	ND	ChIP	[33]			
AP-1	Repressor	HeLa	−1655; −718	EMSA, ChIP	[34]		WT1 [35]	
	Activator	HeLa	−378/+1	ChIP	[36]	HBZ/Sp1 [36]		
AP-2β	Activator	H1299, H322, HBE, WI-38	−43/−18	EMSA, ChIP	[37]			BBR [38]; WT1 [35]
ARID1A	Repressor	OVISE	ND	ChIP	[39]	SIN3A [39]		
β-catenin	Activator	NTera2, SW480, 293T, HCT116, MCF7	−255/+40; −659/−653	EMSA, ChIP	[40,41]	Klf4 [40]		TCF1 [40]
Cbfa1	Repressor	BMSSC	−888/−865; −2815/−2792	EMSA, ChIP	[42]			
c-Myc	Activator	U937, HL60, A549	−242/−237; −34/−29	EMSA, ChIP	[43,44,45]	Sp1 [46];E6 [47]; NFX1-123 [48]; p300 [49];E7 [50]; Max [51]; Ets2 [52]	E6 [53,54,55]; Max [44]; BRCA1/Nmi [56]; NF-κB [57,58]; FOXO3a [59]; MMP-9 [60]; Tax [58]; estrogen [51]; Aurora-A [61]; LMP1 [62]; survivin [63]; PKCθ [64]; SMYD3 [65]; STAGA complex [66]; EGF [67]; CSF1R [68]; GSK3 [69]; DJ-1 [70]; leptin [71]; BDNF [72]; SIRT1 [73]; saquinavir [74]; bile acids [75]; IRF-4 [76]	HIF-1α [77,78,79,80]; BRCA1 [81,82]; cordyceptin [83]; 15d-PGJ2 [84,85]; rosiglitazone [85]; Mad [57,86,87]; ceramide [45]; p53 [88]; Tax [89]; 5-aza-CR [90]; DAC [91]; TMPyP4 [92]; p27KIP1 [93]; genistein [94]; reFIP-gts [95]; 4-hydroxynonena [96]; gambogic acid [97]; butein [98]; wogonin [99]; WT1 [35]; E2F1 [100]; ATO [101]; DADS [102]
COUP-TFII	Repressor	HeLa	−201/+35	EMSA	[103]			
CPSF4	Activator	H1299, A549, H322, WI-38, HBE	−378/+60	ChIP	[104]			
CTCF	Repressor	HeLa, SW480, BJ	+16/+126	EMSA, ChIP	[105]		ROS [106]; TGF-β [107]	Sulforaphane [108]
DEK	Repressor	HeLa	ND	ChIP	[109]		Tax [109]	
E2F1	Repressor	SCC25	−174/−170; −98/−94	EMSA	[110]		TGFβ/Smad3 [111]; PPARα/p16 [112]	
E6	Activator	HK	ND	ChIP	[113]	c-Myc [113]		
EGR-1	Activator	JAR, JEG-3	−281/−273	EMSA	[114]			
	Repressor	CaSki, SiHa	−281/−273	EMSA	[115]			
eNOS	Activator	HUVEC	−949/−935	ChIP	[116]	ERα [116]		
ER81	Activator	293T	+288/+291; +390/+393	EMSA	[117]		HER2/Neu, ERK/MAPK, Ras/Raf [117]	
Estrogen receptor	Activator	MCF7, SiHa, NHK, MDA-MB231, HeLa, OVCA-433, HUVEC	−2677; −949/−935	EMSA, ChIP	[51,116,118]	eNOS [116]	Sp1 [118]; MAPK [119]; leptin [120]	I3C [121]; 15d-PGJ2 [84]; progesterone [122]; miR-498 [120]
ETS	Activator	A549, H1299, MCF7, NIH3T3, A-431, MCF7, ME180	−246/−243; −99/−96; −23/−13	EMSA, ChIP	[52,123,124,125]	c-Myc [52]; EWS [126]	EGF/MAPK [123,124]	PPARγ [127]
	Repressor	U937, K562	−351	EMSA	[125]		WT1 [35]	
GLI1/2	Activator	HT29, 293T	−1226/+438	ChIP	[128]			
GRHL2	Activator	SCC4, NHOK, SCC15	−21/−19	ChIP, PMP	[129,130]			
HIF-1	Activator	ME180, JEG-3, JAR	−165/−158; +44/+51	EMSA, ChIP	[131,132]		LPA [133]; PI3K [133]	
HIF-2	Activator	A498	−165/−158; +44/+51	ChIP	[134]			
	Repressor	U251	−165/−158; +44/+51	ChIP	[134]			
hnRNP D	Activator	NHOK, SCC15, SCC4	−188/−42	ChIP, PMP	[130]			
hnRNP K	Activator	NHOK, SCC15, SCC4	−188/−42		[130]			
Hsp90	Activator	SCC4	−465/−341; −188/+5	ChIP	[135]			
KLF2	Repressor	Kit 225, primary human T cells	+9/+30	EMSA, ChIP	[136]			
KLF4	Activator	FaDu	+18/+77	EMSA, ChIP	[137]			
LSD1	Repressor	HL60	ND	ChIP	[138]			
MAD1	Repressor	WI38, 293T, U937	−243/−238; −34/−29	EMSA, ChIP	[86,139]	Max [140]	Sulforaphane [108]; PPARγ ligands [85]; DADS [102]	miR-202 [140]; MMP-9 [60]; c-Myc [57,86]; miR-202 [140]
Maz	Repressor	HFK	ND	ChIP	[141]		HBx [142]	E6 [141]
MCPH1	Repressor	HeLa	+4/+68	EMSA	[143]			
Menin	Repressor	MCF-7, C33A, HeLa	Sequence-independent	EMSA	[144]	JunD [145]		HBZ [145]
MSH2	Activator	NHOK, SCC15, SCC4	−377/−207	ChIP, PMP	[130]			
MZF-2	Repressor	C33A, SiHa, HeLa, ME100, K562	−687/−680	EMSA	[146]			
NF-κB	Activator	U937, SH-SY5Y, SK-N-MC	−650/−638	ChIP, EMSA	[147,148]		STAT3 [149]; Tax [58,150]; PI3K/Akt [151]	ATO [101]; IκBα [152]; curcumin [148]; pelitinib [153]
NFAT1	Activator	MCF7	−775/−771; −40/−36	ChIP	[154]		Sp1 [154]	
NFX1	Repressor	HFK	−28/−19	EMSA	[48]	Sin3A/HDAC [155]		E6/E6-AP [48]
	Activator	HFK	−28/−19	EMSA	[48]	c-Myc [48]	E6 [55]	WT1 [35]
PAX5	Activator	Raji, Nalm6	+110/+137; +489/+516	EMSA: ChIP	[156]			
PAX8	Activator	LN18, SF268, U87MG	−272/−268; −236/−219; −217/−202; −57/−41	EMSA	[157]			
PITX1	Repressor	A2058	−1366/−1361; −1347/−1342; −1325/−1320	EMSA, ChIP	[158]			miR-19b [159]
PreS2	Activator	HepG2.2.15	−407/−387	EMSA	[160]			
Reptin	Activator	AGS, HGC-27	ND	ChIP	[161]	c-Myc [161]		
RFPL3	Activator	H1299, A549, WI-38, HBE,	ND	ChIP	[162]	CBP [163]		
SNAI1	Repressor	HaCaT, HEK293	−242/−237; −34/−29	ChIP	[80]		TGFβ [80]	
Sp1	Activator	C33A, ME180, SiHa, HeLa	−182; −160; −128; −108; −79	EMSA	[30,46]	CBP [164]; c-Myc [46]	E6 [53,141]; HBZ/JunD [36]; LANA [165,166]; Tax/NF-κB [58]; genistein [167]; survivin [63]; BDNF [72]; PKCθ/NF-κB [64]; SMYD3 [65]; IRF-4 [76]; MCAF1 [168]; NFAT [154]; HBx [169]; HMGA2 [170]	p53 [171]; p73 [172,173]; cordyceptin [83]; 15d-PGJ2 [84]; p27KIP1 [93]; arsenic/ROS [25]; ceramide ([174]; ATO [101]; DADS [102]; E2 [175]; E2F-1 [176]; p16 [177]; butylidenephthalide [178]; indole-3-carbinol [121]; nilotinib/dasatinib [179]; CDDO-Me [180]; homocysteine [181]; triptolide [182]
	Repressor	IMR90, WI38, HFF	−182; −160; −128; −108; −79	EMSA, ChIP	[183]	HDAC [183]; CtBP [184]	TAK1 [185]	E1A [184]
Sp3	Repressor	A549, IMR90, WI38, HFF	−195/−168	EMSA, ChIP	[174,183,186]		Ceramide [174]	
SPT5	Activator	SW620, HT29, Colo320, RKO, HCE8693	−378/+60	ChIP	[187]			
STAT3	Activator	HepG2, MCF-7, DU-145, K562, A172, HS27	−3308/−3300; −1587/−1579	ChIP	[71,167,188,189,190]		miR-21 [191]; leptin [71,188]; GSK3 [69]; HCVc [192]	
STAT5	Activator	ILT-Hod, K562-ADM, K562	−1872/−1864	ChIP	[189,193,194]		IL-2/JAK [193]; EPO [195,196]	
TAL1	Repressor	HeLa	ND	ChIP	[197]	Tax [197]		HBZ/JunD [197]
TEIF	Activator	HeLa, 293, HT1080, 293T	−120/+90	EMSA	[198,199]			MSP58 [199]
USF	Activator	SKBR3, MDA-MB-231, MCF7, OVCAR3, 293T, SKOV3, Wi38, HFF, BJ, lymphocytes, HEK293	−242/−237; −165/−160; −34/−29; +44/+49	EMSA	[200,201]		p300, p38/MAPK [200]; IRF-4 [76]	Truncated USF2 [201]; ATO [101]
	Repressor	OEC-M1, HFK	−242/−237; −34/−29	EMSA, ChIP	[54,202]			
VDR	Repressor	PC3, LNCaP	−2530/−2516	EMSA	[203]	RXR [203]		
WT1	Repressor	293T	−423/−307	EMSA	[204]		PI3K pathway [150]	

**Table 2:** Detail of studies which reported methylation status of human telomerase reverse transcriptase (*hTERT*) promoter proximal region.

Region Tested	Methylation Status	Cell Line/Tissue	Technique to Detect Methylation	Reference
−500 to +50 (72 CpG sites)	Complete	Telomerase-positive (CMV, SUSM-1)	Bisulfite genomic sequencing	[238]
−500 to +50 (72 CpG sites)	Partial	Telomerase-positive (HTB 182, HTB178, CaLu1, CaLu3, CaLu6, HTB57, HCT 116)	Bisulfite genomic sequencing	[238]
−500 to +50 (72 CpG sites)	Unmethylated	Telomerase-positive (A549, HTB183) and telomerase-negative (NHF, MRC-5 p27)	Bisulfite genomic sequencing	[238]
Promoter region	Unmethylated	Telomerase-negative (WI38, HA-1 pre-crisis cell strain, JFCF-6T/5K pre-crisis cell strain, IMR90, BJ fibroblast, telomerase-negative adrenal carcinoma) and telomerase-positive (CT1485)	Methylation-specific PCR-based assay	[243]
Promoter region	Partial or complete	U2OS, GM847, VA13, telomerase-negative breast carcinoma, Co1310	Methylation-specific PCR-based assay	[243]
−441 to +218; with respect to ATG (27 CpG sites)	Hypo- or unmethylated	Telomerase-negative tissue(bladder, brain, heart, kidney, muscle, placenta, skin, testis)	Bisulfite genomic sequencing	[244]
−441 to +218; with respect to ATG (27 CpG sites)	Hypermethylated	Telomerase-positive (MCF-7, A431, HeLa, Co115, HT29, SW480, HS520, SW2, PC3, Saos-2, U2-OS) and two paraffin-embedded fixed tumor tissue (colon, kidney)	Bisulfite genomic sequencing	[244]
−100 and +100	75% to 100% methylated	HeLa, SW480, 8 clones from tumor tissues (breast, bladder and cervix)	Bisulfite genomic sequencing	[105]
−100 and +100	Partial	Telomerase-negative( BJ, HLF)	Bisulfite genomic sequencing	[105]
−80 to −165	Unmethylated	Telomerase-positive (HeLa, SW480) and telomerase negative (BJ, HLF)	Bisulfite genomic sequencing	[105]
−600 region	Complete or partial	Caco-2, HCT116, RKO, SW480, MCF7, MDA-MB-231, MDA-MB-435S, MDA-MB-453, H82, H157, H209, H146, H358, H417, H549, H747, H1299, U1752, DMS53, HL-60, KG-1a, Jurkat, Raji, LCL, VA13	Methylation-specific PCR and bisulfite sequencing	[240]
TSS region	Complete, partial or unmethylated			
Promoter region (27 CpG sites)	Hypermethylated	Downregulated *hTERT* expression in 12 HCC samples and normal tissue	Bisulfite genomic sequencing	[239]
Promoter region (27 CpG sites)	Unmethylated	Downregulated *hTERT* expression in HCC samples	Bisulfite genomic sequencing	[239]
Upstream of the transcription start site (UTSS)	Hypermethylated (5 sites)	Malignant pediatric brain tumor samples	Methylation arrays	[245]
Upstream of the transcription start site (UTSS)	Densely methylated	Neuroblastoma samples	Methylation arrays	[246]

**Table 3:** List of publications reporting *hTERT* promoter mutation frequency in the various cancer types.

Cancer type	References
Atypical fibroxanthomas	[256]
Gliosarcoma	[257,258,259]
Urothelial carcinoma in upper urinary epithelium	[260]
Myxoid liposarcoma	[253,261]
Pleomorphic dermal sarcomas	[256]
Non- invasive and flat papillary urothelial carcinoma	[255]
Urothelial carcinoma with glandular differentiation	[262]
Primary glioblastoma	[253,259,260,263,264,265,266,267,268]
Glioblastoma	[260,269,270,271,272,273,274,275,276,277,278]
Anaplastic oligodendroglioma	[253,259,264,270,272,278]
Metastatic melanoma	[252,279]
Urothelial carcinoma	[253,255,260,262,269,270,271,280,281,282,283,284,285,286,287,288,289]
Basal cell carcinoma	[290,291,292,293]
Squamous cell carcinoma	[290,291,294]
Oligodendroglioma	[259,264,265,268,270,272,276,277,278,295]
Anaplastic thyroid carcinoma	[296,297,298,299,300,301]
Anaplastic oligoastrocytoma	[259,264,278]
Renal Pelvic Carcinoma	[284,302]
Hepatocellular carcinoma	[253,254,260,303,304,305,306,307]
Melanomas	[251,270,271,292,308,309]
Poorly differentiated thyroid carcinoma	[297,299,301]
Oligoastrocytoma	[264,268,276,277,278,310]
Conjunctival melanoma	[311,312]
Primary melanoma	[252,279,313,314]
Tall cell papillary thyroid carcinoma	[315]
Anaplastic astrocytoma	[253,259,264,278,310]
Medulloblastoma	[253,259,316,317]
Ureter carcinoma	[302]
Diffuse astrocytoma	[259,260,264,270,272,277,310,318]
Thyroid carcinoma	[271]
Head and neck squamous cell carcinoma	[276]
Secondary glioblastoma	[263] [259]
CNS lymphoma	[319]
Follicular thyroid carcinoma	[270,296,299,300,320,321,322,323]
Clear cell carcinoma of ovary	[324,325]
Inverted Papilloma of urinary bladder	[287]
Hurthle cell cancers	[297,326]
Malignant pleural mesothelioma	[327]
Solitary fibrous tumor	[261,328]
Acral melanoma	[279,329]
Follicular variant papillary thyroid carcinoma	[315]
Papillary thyroid carcinoma	[270,296,297,299,300,315,320,322,323,330,331,332,333,334,335,336,337,338]
Hemangiopenicytoma	[259]
Pleomorphic xanthoastrocytoma	[259]
Gallbladder	[339]
Adrenocortical carcinoma	[285,340,341]
Renal Cell Carcinoma	[342]
Sinonasal malignant melanoma	[343]
Spitzoid neoplasm	[344]
Malignant peripheral nerve sheath tumor	[261]
Ependymoma	[253,259]
Neuroblastoma	[253,340]
Osteosarcoma	[253]
Meningioma	[253,259,260,345,346]
Anaplastic ependymoma	[259]
Squamous cell carcinoma of the cervix	[253,325]
Synovial sarcoma	[261]
Pilocytic astrocytoma	[259,272]
Neurocytoma	[259]
Testicular germ cell tumor	[347]
Ganglioglioma	[259]
Lung adenocarcinoma	[271]
Gastrointestinal stromal tumor	[260,270,340,348,349]
Esophageal squamous cell carcinoma	[350]
Serous carcinoma of ovary	[325]
Gastric cancer	[339]
Ocular melanoma	[270,311,312,351]
Phaeochromocytoma	[340,341]
Benign thyroid cancer	[270,296,299,321,332,333]
